# The confound of head position in within-session connectome fingerprinting in infants

**DOI:** 10.1016/j.neuroimage.2022.119808

**Published:** 2023-01

**Authors:** Graham King, Anna Truzzi, Rhodri Cusack

**Affiliations:** a Trinity College Institute of Neuroscience and School of Psychology, Rm 3.22 Lloyd Building, Trinity College Dublin, Dublin 2, Ireland; b Neonatology Department, The Rotunda Hospital, Parnell Square, Dublin 1, Ireland

**Keywords:** Connectome, Infant, Preterm, Fingerprint, Head coil, Functional MRI, BOLD, blood oxygen level dependent, dHCP, Developing Human Connectome Project, DWMA, diffuse white matter abnormalities, Fc, functional connectivity, fMRI, functional MRI, FWD, framewise displacement, OFC, occipital-frontal circumference, PMA, post-menstrual age, ROI, region of interest, rsfMRI, resting state fMRI, RSM, representational similarity matrix, RSNs, resting state networks, SD, standard deviation, SNR, signal-to-noise ratio (a.k.a. tSNR temporal signal-to-noise ratio), SNR-coil, estimated SNR for each point in the head coil space, SNR-individual, real SNR calculated from the participant's functional timeseries, SNR-group, SNR-coil values resliced back into individual participant's functional space and re-sampled using Schaefer ROIs, TEA, term equivalent age

## Abstract

Individuals differ in their functional connectome, which can be demonstrated using a “fingerprinting” analysis in which the connectome from an individual in one dataset is used to identify the same person from an independent dataset. Recently, the origin of these fingerprints has been studied by examining if they are present in infants. The results have varied considerably, with identification rates from 10 to 90%. When fingerprinting has been performed by splitting a single imaging session into two split-sessions (within session), identification rates were higher than when two full-sessions (between sessions) were compared. This study examined whether a methodological difference could account for this variation. It was hypothesized that the infant's exact head position in the head coil may affect the measured connectome, due to the gradual inhomogeneity of signal-to-noise in phased-array coils and the breadth of possible positions for a small infant head in a head coil. This study examined the impact of this using resting state functional MRI data from the Developing Human Connectome Project second release. Using functional timeseries, fingerprinting identification was high (84-91%) within a session while between sessions it was low (7%).Using *N* = 416 infants’ head positions, a map of the average signal-to-noise across the physical volume of the head coil was calculated and was used (independent group of 44 infants with two scan sessions) to demonstrate a significant relationship between head position in the head coil and functional connectivity. Using only the head positions (signal-to-noise values extrapolated from the group average map) of the independent group of 44 infants, high identification success was achieved across split-sessions (within session) but not full-sessions (between sessions). Using a model examining factors influencing the stability of the functional connectome, head position was seen as the strongest of the explanatory variables. We conclude within-session fingerprinting is affected by head position and future infant functional fingerprint analyses must use a different strategy or account for this impact.

## Highlights

• Identifying functional fingerprints in infants has mixed results (10–90% ID rates).
• An infant's exact position in the head coil affects the measured connectome.
• Within-session (split segment) fingerprinting is strongly affected by head position.
• Infant head coil design should aim to mitigate head position induced artifacts.
• Future across-session methods should control for head position induced artifacts.

## 1 Introduction

In recent years a growing literature regarding the infant functional connectome has developed. To some degree, the resting state networks (RSNs) found in adults have also been found in infants (Doria et al., 2010; Fransson et al., 2007; Gao et al., 2016, 2015; Linke and Cusack, 2015; Smyser et al., 2010). In a recent large cohort of term born infants scanned at 37 – 43 weeks post-menstrual age (PMA), an adult-like topology was found in RSNs in the primary sensory, motor, visual, and auditory cortices (Eyre et al., 2021), while in association RSNs, maturation in functional connectivity (Fc) was found across the age span. In preterm infants, RSNs have been identified as young as 29 weeks PMA (Doria et al., 2010) and even at 26 weeks PMA (Smyser et al., 2010).

Preterm birth can affect the functional connectome. Studies have shown various network-specific differences in resting-state functional MRI (rsfMRI) connectivity between very preterm and term infants when scanned at term equivalent age (Ball et al., 2016; Smyser et al., 2016). Further, Eyre et al. found that preterm birth is associated with decreased functional connectivity at term across all defined RSNs in a dose-dependent manner (Eyre et al., 2021). In addition, neuro-pathologies associated with the preterm neonate (such as post-hemorrhagic or moderate/severe diffuse white matter abnormalities, DWMA) have been shown to result in reduced functional connectivity at term equivalent age (TEA) (He and Parikh, 2015; Smyser et al., 2013).

In addition to these differences between groups, substantial individual variation in functional connectomes has been found in children and adults. In the Human Connectome Project a large cohort of adults was scanned twice, and the connectome from a person in the first scan could be used to find that person among the connectomes from the second scans with high accuracy – a procedure that has been dubbed “fingerprinting”. Fingerprinting shows that a significant proportion of individual variance in functional connectivity is trait-like, or intrinsic to the participant and constant across time, and only weakly impacted by the modulations in brain state that occur across sessions (Finn et al., 2015; Miranda-Dominguez et al., 2014). Importantly, these individual differences in brain connectivity have been shown to be predictive of individual differences in behavior in adults (Finn et al., 2015; Ousdal et al., 2020). This suggests that fingerprinting captures a functionally important component of the connectome.

Recently, a number of groups have used fingerprinting to test whether stable individual differences in the functional connectome are present in infants (Ciarrusta et al., 2021; Dufford et al., 2021; Hu et al., 2021; Q. Wang et al., 2021; Y. Wang et al., 2021). The results are quite disparate. A cohort of 31 asleep preterm infants scanned in two rsfMRI sessions separated by a few weeks had an identification rate of just 3/31 (10%) (Ciarrusta et al., 2021). In another cohort of 30-40 term infants scanned at 1 month and 9 months the identification rate was 50–65% (Dufford et al., 2021). A rsfMRI study of 239 asleep infants (aged 39 – 59 weeks PMA) reported moderate-to-good reliability (intraclass correlation 0.40–0.78) (Y. Wang et al., 2021). Finally, a recent study of 40 healthy term infants scanned asleep at 1 week of age, found a connectome identification rate of 100%, leading the authors to report that the connectome fingerprint is already present in neonates ( Q. Wang et al., 2021).

It is believed that a methodological difference may be largely responsible for the disparity of these results, namely whether fingerprinting is calculated by comparing two different scanning sessions or is derived from within a scanning session. A straightforward way to conduct fingerprinting within a session, for example, is to compare the connectome derived from the first half of a rsfMRI session with the connectome from the second half of the same session (Q. Wang et al., 2021). Supporting the importance of this methodological difference, in a study using both methods, the reported across-session identification was much lower (13–20%) than within-session identification (50–65%) (Dufford et al., 2021).

Two explanations were considered for why across-session identification was lower than within-session identification. One is that the connectome may be unstable in the infant brain. The extent to which the functional connectome is reproducible across time at the participant level (i.e., “stable”) is an area of much interest in recent years. It has been shown to be stable across ‘days to weeks’ in adults (Finn et al., 2017, 2015; Horien et al., 2019; Kaufmann et al., 2018; Miranda-Dominguez et al., 2014; Vanderwal et al., 2017) with identification accuracies up to approximately 90%. Further, the functional connectome has been seen to be stable across ‘years’ in children/youths and adults (Jalbrzikowski et al., 2020; Miranda-Dominguez et al., 2018). However, it might be that in the highly plastic and rapidly changing infant brain, connectomes are unstable within an individual. Across the 8-month gap between the two scans in the Yale group study (Dufford et al., 2021), there are large changes in functional brain organization. Synaptic connections change rapidly through the first year (Huttenlocher and de Courten, 1987). Myelination commences at approximately 20 weeks PMA and rapidly progresses over the first 2 years of infant life (Barkovich, 2005); and neurovascular changes in infants occur over a matter of days, which will affect the functional MRI (fMRI) signal (Kozberg and Hillman, 2016). The instability of the infant connectome may lead to lower across-session than within-session fingerprint identification rates.

In contrast to the idea that the infant connectome is unstable is a recent publication from the Baby Connectome Project (Hu et al., 2021) which has reported higher identification rates suggesting that infant connectomes are unique and stable over months. In this study 104 term born infants were scanned at least twice at different sessions (between ages 16 – 874 days) resulting in 806 longitudinal rsfMRI scans. The study used a fine-grained infant-specific mid-cortical functional parcellation map with 602 cortical regions-of-interest (ROIs) (higher parcellation is known to improve connectome distinctiveness). Importantly this study used feature selection-based identification by estimating the differential capability of an edge by its standard deviation (SD) across subjects. The peak threshold value for SD was found to be 0.95 – 0.98 for optimum identification rate and using this optimum threshold the correct identification rate was approximately 66% across the group (in contrast to 47% without feature selection) (Hu et al., 2021).

The second explanation considered was that within-session fingerprinting may be affected by an artifact of the acquisition process, the head position in the MRI head coil. Head coils with many receiving coils are used for fMRI to provide high signal-to-noise, but these are gradually inhomogeneous across the volume of the head coil, with the signal closest to the receiver elements a factor of 2 – 3 times higher than in the center of the coil (Ghotra et al., 2021; Hughes et al., 2017). Despite using the same head coil, different infants will have different head positions due variable initial placement of the head, followed by idiosyncratic application of the head cushions on each side of the infant's head. As a result, head position will be more similar within a session compared to between different sessions. If the signal-to-noise (SNR) field was completely homogenous, or completely heterogenous, head position would not lead to SNR differences. However, as the SNR field has a gradual inhomogeneity with smooth changes (see Figure 3) then head position may lead to substantial modulations in SNR. In this case a small amount of head position change (common within session, i.e. across split-session analyses) will lead to only small changes in SNR, but larger differences in head position (common between sessions, i.e. across full-session analyses) will lead to larger differences in SNR (and resulting functional connectivity pattern). As a result, the similarity of the functional connectome may be more similar during split-session analyses due to this similarity of head position (SNR artifact). Put in another way, within session fingerprinting may be decoding head position in the MRI scanner, rather than, or in addition to, capturing individual differences in the brain connectivity.

The goal of this manuscript was to investigate these two possibilities. This study tested the hypothesis that infant's head position affects signal-to-noise, and that this in turn affects the measured connectome. It further tested the hypothesis that within-session fingerprint identification is inflated by differences in signal-to-noise related to session-to-session differences in head position.

## 2 Methods

### 2.1 Cohort details

Open data from the Developing Human Connectome Project (dHCP) second release (http://www.developingconnectome.org/data-release/second-data-release/) was used. Rs-fMRI data had been obtained using multiband x9 acceleration echo-planar imaging (TE/TR: 38/392 ms) providing 2300 volumes with an isotropic resolution of 2.15 mm. fMRI data was pre-processed as per the dHCP Data Organization (http://www.developingconnectome.org/data-release/second-data-release/release-notes/) and as stipulated in the dHCP publication (Fitzgibbon et al., 2020)

The main steps of the pre-processing pipeline included (Fitzgibbon et al., 2020):

- Estimation of the susceptibility distortion field
- Registration of blood oxygen level dependent (BOLD) images with native T2 space and the neonatal atlas space
- Susceptibility and intra- and inter-volume motion correction (slice-to-volume motion correction, dynamic susceptibility distortion correction, and estimation of motion nuisance regressors) (Andersson et al., 2017)
- Denoising: regression of signal artifacts related to head motion, cardiorespiratory fluctuations and multiband acquisition, together with single-subject ICA noise bespoke components identified with the FMRIB's ICA-based Xnoiseifier (FIX) (Salimi-Khorshidi et al., 2014).

Following pre-processing and quality control 512 fMRI sessions were included in the dHCP data release (416 participants with 1 scan, 48 participants with 2 scans). The 416 participants with one scanning session included 34 who were born preterm and 382 who were born term. The infants with two rsfMRI scanning sessions (*n* = 48) were born between 25 and 37 weeks post-menstrual age (PMA) and scanned first (preterm session) between 29 and 37 weeks PMA (median 34.9 weeks PMA, IQR 2.5 weeks) and again (term session) at term age (median 41 weeks PMA, IQR 2.1 weeks).

### 2.2 Registration and Region-of-Interest analysis

A ROI analysis was carried out using the Schaefer 400 functional parcellation (Schaefer et al., 2018) in the individual infants’ functional spaces. This functional parcellation has been shown to perform similarly to the Glasser multi-modal parcellation when evaluated using the ‘distance-controlled-boundary-coefficient’ (a measure that allows for comparison of parcellation with different spatial resolutions) (Zhi et al., 2021). The adult T1 MNI-152 template was used facilitate registration of the Schaefer-400 to the dHCP 40-week infant template (Schuh et al., 2018), following the procedure described in (Cabral et al., 2020). As the infant skull is very close to the cortex it was noted that the adult cortex registered to this and as a result the skull was removed from infant images by taking background tissue, label 4 in the dHCP 40-week atlas (https://gin.g-node.org/BioMedIA/dhcp-volumetric-atlas-groupwise), thresholding at 1000, inverting the mask, and applying it to the dHCP T1 infant template. It was also noted that registration led to the inferior visual cortex being dragged down, likely due to the small infant cerebellum, and so the cerebellum was masked out in the infant and adult templates. To do this in the adult template a mask of the cerebellar region 95-120 (AAL3 template 62 from https://www.oxcns.org/aal3.html) was created, inverted, and applied to the T1 adult template. The infant cerebellum was masked using tissue type 6 (dHCP 40-week atlas), thresholding by 300, inverting, and applying the mask to the T1 40-week template.

The transformation from the adult T1 MNI-152 template (cerebellum stripped) to the dHCP 40-week infant template (skull and cerebellum stripped) was carried out with ANTS (http://stnava.github.io/ANTs/). This transformation was then applied to the MNI-space Schaefer atlas. The FSL transform (provided by the dHCP release) was used to transform Schaefer-400 from the dHCP 40-week atlas space to the individual infants’ functional spaces.

#### 2.2.1 Quality control

Quality control showed that there were artifacts in rsfMRI images from some participants leading to signal loss in some ROIs. For each session, ROIs with a signal intensity less than 3 standard deviations below the mean across ROIs were defined as outliers. ROIs which met this outlier definition across >10% of the sessions were taken as ROI deviants and were removed, leaving 387 ROI (from original 400) for subsequent analysis (ROIs removed included bilateral prefrontal cortex lateroventral areas, limbic-temporal pole areas, and visual centers-extra striatal areas).

A quality control of the FSL transform (used in this analysis to transform Schaefer-400 from the dHCP 40-week atlas space to the individual's functional space) was also conducted. This quality control involved visualizing the overlay of the Schaefer-400 over both the functional brainmask and the mean BOLD image of the timeseries while in the individual functional space. This was done for all 96 sessions (48 participants with both preterm and term sessions from the dHCP Data Release 2.0, 2019). This FSL transform was adjudged to have been ineffective (in at least 1 of the 2 sessions) for 4 out of the 48 participants. These 4 participants were removed leaving the cohort with 44 participants remaining (88 sessions).

Motion can have a strong effect on rsfMRI, and even sleeping infants occasionally move in the scanner (Cusack et al., 2018). To quantify this, the mean framewise displacement in a session was used (Power et al., 2012).

### 2.3 Connectivity and connectome stability calculations

#### 2.3.1 First order (across time) analysis

Segments of the functional timeseries were derived from both full sessions (full timeseries, 2300 volumes, 15 mins) or split-sessions (partial timeseries). To create a split-session segment a session was split into two smaller segments by removing 60 seconds (154 volumes) from the middle of a session's timeseries and labelling the two remaining sections as ‘split-sessions’ (Q. Wang et al., 2021).

Each segment was filtered to the functional connectivity (Fc) frequency range (0.1 – 0.01 Hz) using nilearn (https://nilearn.github.io/modules/generated/nilearn.signal.clean.html). For each segment, the functional connectivity of every pair of ROIs was calculated using Pearson correlation between their timeseries to yield the connectome.

#### 2.3.2 Second order (across segment) analysis

For correlation across segments (full or split-sessions) the similarity of the resulting ROI by ROI connectome for each participant's first segment was then compared to each participant's second segment using Spearman correlation (across-segment correlation). Following calculation of functional connectivity (Pearson correlation across the timeseries) it is well recognized that the resulting values may not be normally distributed (Kriegeskorte et al., 2008) and so a second order Pearson correlation would be inappropriate. As a result, Spearman correlation was used to assess the higher order (across full-sessions or across split-sessions) comparison of functional connectivity (ROI based edges). The within-participant Spearman correlation gives an indication of the stability of the connectome for that participant (a.k.a. ‘connectome stability’).

#### 2.3.3 Connectome stability based identification (fingerprinting)

The connectome-based identification (ID) method followed that suggested by Finn et al. (2015). Identification was performed across pairs of segments with the first order connectivity matrix from one segment the ‘target’ and the other the ‘database’. The target and database had to be selected from different timepoints (either across full-session segments, or, across split-session segments). Similarity was defined as the Spearman rank correlation coefficient between the various pairs of target and database matrices. If the target and database pair with the highest similarity originated from the same participant, this was considered a ‘match’. The percentage of participants where identification was correctly predicted was calculated for the total number of participants (44 participants). This ability to ‘match’ (highest similarity with self) is thought to be due to a connectome ‘fingerprint’ whose stability allows correct identification (fingerprinting).

### 2.4 Using signal to noise to examine the Impact of Head Position

Traditionally signal-to-noise (SNR) is calculated from the functional timeseries of a session. Of note, it is recognized that ICA denoising (dHCP pre-processing pipeline) artificially inflates the SNR of the functional timeseries shipped with this Data Release 2.0 (Fitzgibbon et al., 2020). Despite this fact, this study used the minimally pre-processed data to determine both the real SNR and the SNR resulting from head position alone (see explanations of both of these below).

#### 2.4.1 Estimation of signal to noise using functional timeseries

SNR was calculated as the mean BOLD signal divided by the detrended standard deviation (SD) (Friedman and Glover, 2006) for the timeseries at each voxel in native space. Detrending was carried out using nilearn (https://nilearn.github.io/dev/modules/generated/nilearn.image.clean_img.html). For segments consisting of full-sessions, the SNR was calculated from the full timeseries. For segments consisting of split-sessions, the SNR was calculated from each of two split timeseries (leaving out 60 secs from the middle of the session as described previously for functional connectivity). The resulting segment-specific SNR map allowed extraction of edge values using the Schaefer 40-week atlas in the individual ‘s space. These SNR values were labelled as ‘SNR-individual’ (to discern them from ‘SNR-group’ values, as described below).

#### 2.4.2 Estimation of signal to noise using head location

The signal-to-noise ratio (SNR) across the head coil was calculated as follows:

• A group of 416 participants with a single session (34 preterm, 382 term) was used. This group was independent of the 44 participants with two sessions (preterm and term sessions).
• The SNR for each individual was calculated from the fMRI timeseries at each voxel in native space as the mean signal across time divided by the detrended standard deviation (SD) across time (Friedman and Glover, 2006)
• Our goal was to quantify SNR variation across the coil. The internal structure of the brain (e.g., the contrast between grey and white matter) was not present in the SNR maps and instead the well-established multi-channel receive coil sensitivity profile (Wiggins et al., 2006) was dominant (i.e., lower signal further from the coil). An exception to this was round the edge of the brain, where there was a thin band of lower SNR values, consistent with CSF signal. As a result, the SNR images were masked with a brain mask that had been eroded by a 1 voxel (2.1mm).
• Each individual's fMRI data had an accompanying affine transformation matrix, comprising the translation, rotations, and scaling, to map the voxel coordinates into the frame of reference of the MRI scanner (i.e., mm for x, y and z relative to the isocenter). Using this, the SNR maps from each individual were resliced to a common 1 mm isotropic grid into the scanner's space using the MNI152_T1_1mm atlas shipped with FSL (Fonov et al., 2009).
• These individual maps were averaged across the 416 participants (using a soft mean), giving an estimated SNR for each point in the head coil (termed ‘SNR-coil’). Supplementary Figure S 1 shows the proportion of the 416 participants used to calculate the soft-mean for each voxel of the SNR-coil map.
• The group average map (SNR-coil) was then used to predict the SNR for each ROI of each individual, from the position and orientation of their head. These predicted SNR values, labelled here as ‘SNR-group’ values, represent the SNR values resulting from the position, orientation, and shape of the head relative to the head coil.

For full-session segments (across session analysis) SNR-coil was resampled to the individual's native space and the values extracted in the ROIs from the Schaefer 40-week atlas in that individual's space.

For split-session segments (within session analysis) two head positions were chosen (positions at volume 537 and volume 1763 of the functional MRI timeseries for a session). Volume 537 represents the head position in the middle of the first segment, the volume that FSL MCFLIRT would realign to if working on only the first split of the session. Volume 1763 similarly represents the head position in the middle of the second split-session segment. The 6 rigid-body motion parameters (dHCP Data Release, Quality Control data), 3 translations and 3 rotations, represent the head position relative to the initial volume (volume 0). These 6 motion parameters for each head position were translated to FSL – FLIRT format and the resulting matrices were used to re-orient the individual's Schaefer 40-week atlas, using FSL FLIRT, to the two head positions in their individual participant functional space. For the 44 participants with two sessions, SNR-group values were extracted from the SNR-coil map (using FSL fslmeants) for both of the two reoriented Schaefer 40-week atlas head positions for each of 88 sessions (creating 176 SNR-group value matrices for all 176 split-sessions).

#### 2.4.3 Fingerprinting – a comparison of SNR-group and SNR-individual

In order to compare their ability to mediate fingerprinting this study assessed whether SNR-group or SNR-individual in one segment (full-session/split-session) better predicted functional connectivity in another segment. This analysis can be found in the Supplementary Materials (Equation S1). Figures S2 shows that, for both full-session and split-session segments, SNR-individual had less predictive power for individual differences than SNR-group. This suggests that coil sensitivity dominates the predictive power of SNR, and that this is best estimated using group-average data. In this regard, it was decided to use the SNR-group measure for further analyses in this manuscript.

### 2.5 Head position and functional connectivity

The relationship between the predicted SNR (SNR-group) values and functional connectivity (Fc) was explored using linear regression. For each of the 74,691 edges, a linear model across the 416 participants with one session was fitted Eq. (1). The rational for using a linear regression model ‘for each edge’ (linear regression across participants) was to examine the relationship between functional connectivity and SNR (SNR-group) due purely to head position. An edge represents the connection between two ROIs, and while that connection is consistent in terms of anatomical regions, the position of the edge in the head coil space changes with head position. This study examined whether a change in head position in the head coil space (SNR-group value) may or may not result in a change in functional connectivity at that same edge.

(1) $$c_{ij}^{\left(p\right)}=\beta_{ij}s_{i}^{\left(p\right)}s_{j}^{\left(p\right)}+a$$

where

p is the participant number
$c_{ij}$ is the functional connectivity of edge connecting regions *i* and *j*
*s_i_* and *s_j_* are the SNR-group of regions *i* and *j*
$\beta_{ij}$ is the slope of the relationship for this edge
a is a constant

The statsmodels ordinary least squares linear regression function was used (https://www.statsmodels.org/stable/generated/statsmodels.regression.linear_model.OLS.html). Values for functional connectivity and SNR-group were standardized (z-score). Non-zero β values would indicate a consistent relationship between ‘SNR due to head position’ and functional connectivity. A permutation test was used to test this. As the regression model is not entirely independent of the input values (the same 416 sessions were used to define the SNR-coil) it was decided to cross-validate in an independent group of 44 preterm participants each with two scans (88 sessions).

### 2.6 Head position and fingerprinting

Firstly, to examine the similarity of SNR-group values across participants and across segments (full-session or split-session), the Spearman rank correlation coefficient between the various pairs of target and database SNR-group matrices was used (similar to 2.3.3 above). Again, if the target and database pair with the highest similarity originated from the same participant, this was considered a ‘match’ and the percentage of participants where identification was correctly predicted was calculated (using only these head position derived SNR-group values).

Secondly, to quantitatively compare the impact of head position (SNR artifact) with the functional connectivity signal that drives fingerprinting, both the functional connectivity and SNR (SNR-group) from the first segment were entered as standardized regressors to predict the functional connectivity in the second. This was implemented using a statsmodels ordinary least squares multiple linear regression function.

The standardized regression coefficients this yielded were used to assess predictor strength. The regression model took the form Eq. (2):

(2) $$c_{ij}^{\left(p,2\right)}=\beta_{ij}^{SNR}s_{i}^{\left(p,1\right)}s_{j}^{\left(p,\;1\right)}+\beta_{ij}^{FC}c_{ij}^{\left(p,\;1\right)}+a$$

where

$c_{ij}^{\left(p,e\right)}$ is the functional connectivity of the edge between regions i and j for participant p and segment number e (session/split)
$\beta_{ij}^{SNR}$ is the standardized regression coefficient of SNR-group for the edge
$s_{i}^{\left(p,e\right)}$ is the SNR-group value of region i for participant p and segment e
$\beta_{ij}^{FC}$ is the standardized regression coefficient of functional connectivity for this edge
a is a constant

SNR-group and functional connectivity were both standardized to obtain z-scores, so that the beta parameters became comparable and indicative of the variance explained. The focus of fingerprinting is on individual differences, and so a separate regression model was conducted for each edge to model the variance across the 44 participants. Regression was repeated for segments comprising two full-sessions and for segments comprising two split-sessions. It was repeated in both directions (the dependent variable ($c_{ij}$) being from segment 1 or segment 2).

### 2.7 Factors impacting connectome stability

#### 2.7.1 Individual explanatory variables

For the 44 participants with two sessions, the relationship between connectome stability and one other individual explanatory factor (head size, inter-session interval, and motion respectively) was examined using a scatter plot and Pearson correlation (with confidence interval and p-value). Methods and results of these respective individual correlations are described in Supplementary Sections S3.1 through S3.3.

#### 2.7.2 Multiple explanatory variables

To assess the degree to which head position (SNR-group) impacts connectome stability (dependent variable) and compare it to other explanatory variables, a statsmodels ordinary least squares multiple linear regression model was used for full-sessions Eq. (3) and split-sessions Eq. (4), respectively.

The 44 participants with two sessions were included in this model. In all instances the explanatory variables were standardized (z-score) prior to inputting into the regression model.

Across full-session segments Eq. (3) the dependent variable was connectome stability across full-sessions (44 values). The five (i=5) explanatory variables considered included: mean SNR-group (mean across the two full-sessions); mean framewise displacement (FWD) (mean across the two sessions); inter-session interval (weeks); age at first session (post-menstrual age); and mean occipital-frontal circumference (OFC) (mean across the two sessions).

(3) $$s^{p}=\sum_{i=1}^{5}\beta_{i}x_{i}^{p}+a$$

where,

i is the number of explanatory variables
$s^{p}$ is connectome stability for participant p
$x_{i}^{p}$ is explanatory variable number i for participant p
$\beta_{i}$ is standardized regression coefficient number i
a is a constant

Across split-session segments Eq. (4) the dependent variable was connectome stability across split-sessions (88 values). The two (i=2) explanatory variables considered were mean SNR-group (mean across the two split-sessions) and mean FWD (mean across the two split-sessions).

(4) $$s^{\left(p,\;e\right)}=\sum_{i=1}^{2}\beta_{i}x_{i}^{\left(p,e\right)}+a$$

where,

e is session number, where e∈{1,2}
i is the number of explanatory variables$s^{\left(p,e\right)}$ is connectome stability for participant p session e
$x_{i}^{\left(p,e\right)}$ is explanatory variable number i for participant p session e
$\beta_{i}$ is standardized regression coefficient number i
a is a constant

### 2.8 Ethics statement

Data was obtained from the dHCP Data Release 2 (2019) with permission. In order to protect data participants, these open data have been pseudonymized and subjected to post-collection processing (data processing) at source to remove any data elements that could otherwise be reconstructed and/or processed to reveal facial features of the data participants. Analysis of this data was approved by the Trinity College Dublin School of Psychology Research Ethics Committee (ID: SPREC082021-02).

## 3 Results

### 3.1 Functional connectivity and fingerprinting

Like Dufford et al. (2021), it was seen that fingerprint identification was much higher across split-session segments than across full-session segments (Dufford et al., 2021). Across full-session segments only 3 of 44 (7%) of infants’ connectomes ranked highest (connectome correctly identified across sessions), while within a session (across split-session segments) 40/44 (91%) of preterm and 37/44 (84%) of term infants ranked highest (Table 1).

**Table 1:** Functional connectome: stability across versus within sessions.

	Across-sessions (88 full-sessions)	Within-sessions (176 split-sessions)	
		Preterm (88 splits)	Term (88 splits)
**Within-participant correlation (mean)**	0.17	0.37	0.46
**Between-participants (shared) correlation (mean)**	0.13	0.07	0.18
**Identification Success**	3/44 (7%)	40/44 (91%)	37/44 (84%)

**Fig. 1:**
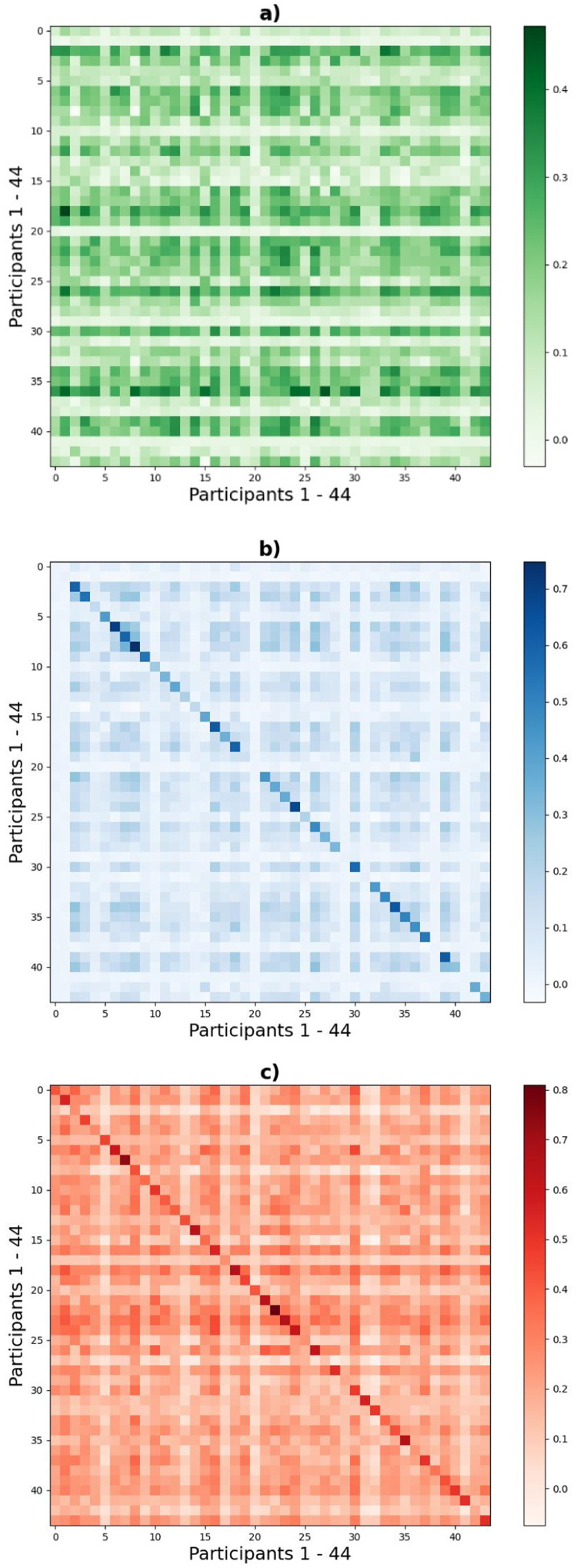
Representational Similarity Matrices (44 participants with two sessions). Second order Spearman correlations of functional connectivity: (a) across sessions, (b) across split-segments at preterm session, (c) across split-segments at term session.

Fingerprinting might fail across sessions either because an individual's connectome is unstable, or because relatively, different people have a more similar connectome across sessions. To differentiate these possibilities, within-participant and between-participant connectome similarity was examined, using Spearman (second-order) correlations (Table 1). Across full-session segments the within-participant correlation (connectome stability) was low, with the mean of 44 participants (r = 0.17) only slightly higher than the mean of the across-participant (shared) correlation (r = 0.13). The 44 × 44 representational similarity matrix (RSM) had no obvious leading diagonal (no significant correlation within a participant across the two full-sessions) (Fig. 1). In contrast, Spearman correlations within a participant were much higher when comparing split-session segments (within a session) at both preterm age and term age (Table 1 and Fig. 1). This shows that within-session fingerprinting is more effective because the similarity of the connectomes within a participant are much higher.

Overall, this picture of strong self-identification within a session (across split-session segments) but weak self-identification across sessions (across full-session segments) is consistent with either of the two explanations – it might be because infant connectomes are unstable over weeks, and/or because of various artifacts contributing towards high within-participant correlation during within session analysis.

### 3.2 Head position and functional connectivity

**Fig. 2:**
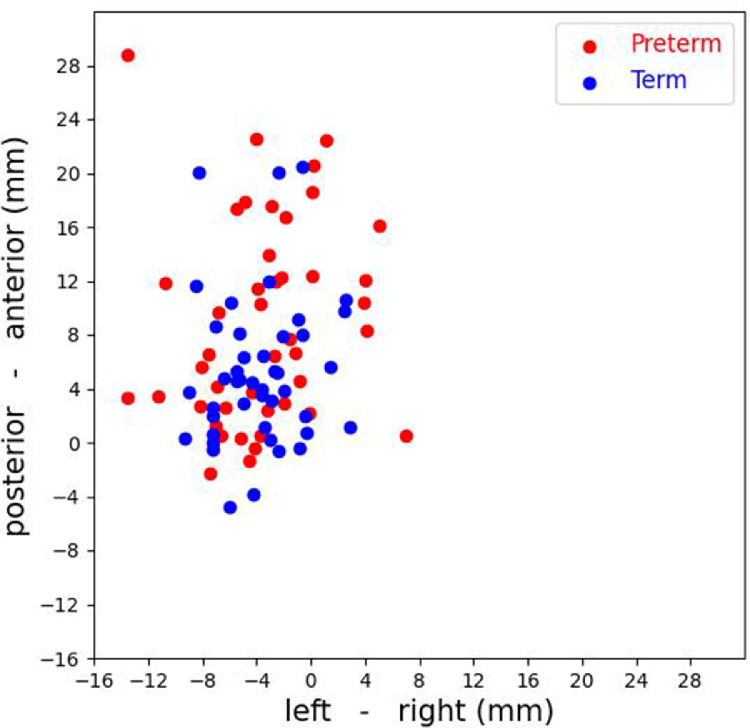
Centroids of brainmasks in scanner space for 44 participants (preterm & term sessions). For a 20mm displacement in either direction, this could result in an approximate 50% decrease in SNR values in the dHCP dedicated neonatal head coil (values derived from data on phantom analysis by Hughes et al. (2017).

To demonstrate the variability of head position for the 44 participants, FSL fslstats was used to calculate the centroid (‘center of gravity’) of the functional brainmask for a session. This gave the centroid in mm coordinates (x, y, z scanner directions). To illustrate the variability in centroid position the centroids (x, y coordinates) for both preterm and term sessions (44 participants) were plotted on a 2-dimensional scatter plot (Fig. 2). This plot shows a clear distribution of centroids with variability in the posterior - anterior direction (range: -5mm to +29mm) and the left - right direction (range: -14 to +7mm). There was more variability in centroid position in the preterm session compared to the term session.

**Fig. 3:**
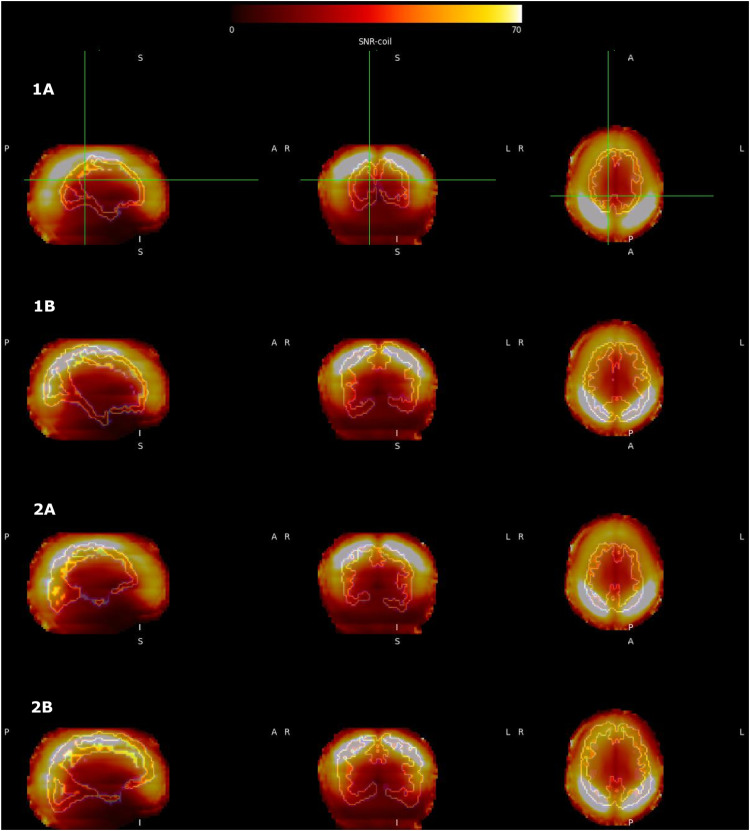
Impact of Head Position - *Scanner Frame of Reference*: Schaefer 40-week atlas resliced from individual space to MNI152 reference and overlayed on SNR-coil. Two example preterm participants with two sessions were chosen: (1A) Participant 1 at 29.9 weeks PMA (1B) Participant 1 at 38.4 weeks PMA. (2A) Participant 2 at 35.8 weeks PMA (2B) Participant 2 at 42.1 weeks PMA.

To test the hypothesis that differences in SNR due to head position affect connectome fingerprinting, an estimate of the SNR across the volume of the coil (SNR-coil) was derived and then used to estimate each individual's SNR based on location of their head (termed ‘SNR-group’). This was illustrated for two participants in the scanner frame of reference (Fig. 3) and in the brain frame of reference (Fig. 4).

**Figu. 4:**
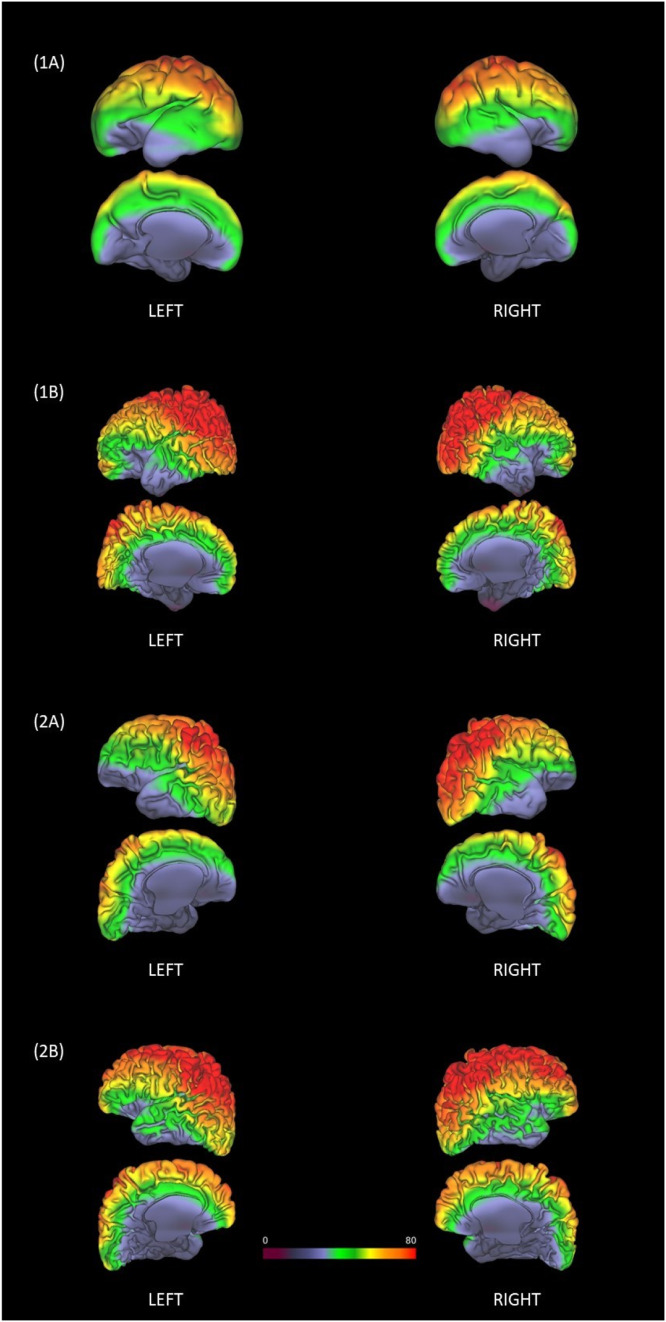
Impact of Head Position - *Brain Frame of Reference*: SNR-coil resliced to individual space and mapped to mid-cortical thickness surfaces (T2w) (Left + Right). Two example preterm participants with two sessions were chosen: (1A) Participant 1 at 29.9 weeks PMA (1B) Participant 1 at 38.4 weeks PMA. (2A) Participant 2 at 35.8 weeks PMA (2B) Participant 2 at 42.1 weeks PMA.

The scanner frame of reference (Fig. 3) depicts the individual's Schaefer 40-week atlas resliced into MNI152 reference space and overlayed onto the SNR-coil. Head position and distance from the head coil directly are seen to influence the pattern of SNR across the brain. Using the brain frame of reference (Fig. 4), the SNR-coil was resliced to individual space and mapped to the mid-cortical thickness surface (T2w) using Connectome Workbench. Based on position in the head coil, it can be seen that the mid-cortical thickness surface has a variable pattern of SNR across the various sessions (preterm and term examples).

**Fig. 5:**
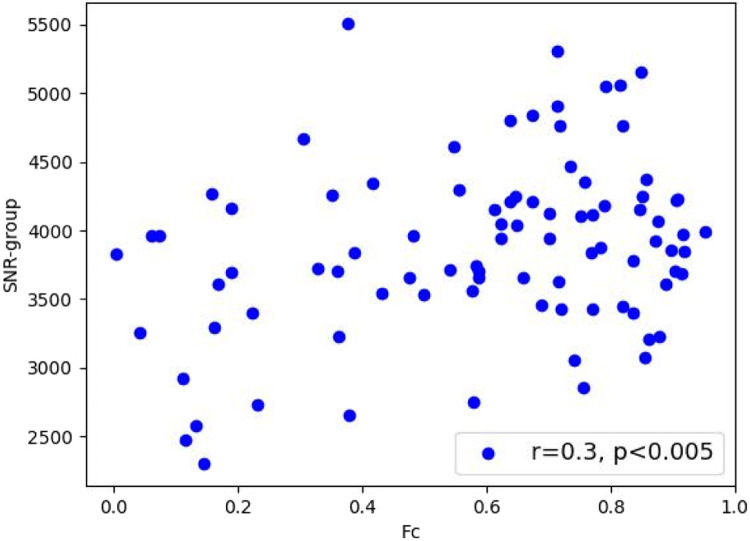
Across 88 sessions (44 participants with 2 sessions) SNR-group values covary with Fc (Schaefer ROI edge 174–175).

To demonstrate how SNR covaries with Fc, a pair of parcels (ROIs) from the Default Mode Network (DMN) were chosen. Within the DMN regions one pair was selected based on having the highest mean functional connectivity across the 88 sessions (Mean r = 0.595). This was the edge between the inferior parietal lobe (IPL) and the dorsal pre-frontal cortex (PFCd) (Schaefer regions 174 and 175) on the left hemisphere. Across the 88 sessions (44 participants with 2 sessions) functional connectivity was plotted against SNR-group values for this edge (Fig. 5). The relationship showed that there was a positive correlation between Fc and SNR (r = 0.3, 95%CI 0.09-0.48, *p* < 0.005).

Next, using ordinary least square regression across 416 participants for each edge, the relationship between SNR-group and functional connectivity was examined. For each of the 74,691 edges the *β* (slope) for the fit was calculated across all 416 participants and a histogram of Beta values was plotted (*Supplementary Figure S6*). The one-sample t-test showed the *β* values were significantly different from zero (*p* < 0.0001) indicating a significant relationship between predicted (based on head position) SNR values and functional connectivity.

In this analysis, although a significant relationship was seen between SNR-group values and functional connectivity across the 416 participants, there was a slight possibility of circularity due to the fact that the same data was used to both a) generate the group average ‘SNR-coil’, and, b) compare the relationship between SNR-group and functional connectivity values in the same cohort. In light of this, this significant relationship was cross-validated in the independent sample of 44 participants (each with both preterm and term sessions) whose sessions’ data were not used in the calculation of SNR-coil. Using Eq. (1) for the 88 independent sessions, the relationship between SNR-group and functional connectivity was re-examined. The histogram of 74,691 slopes (*β*) (*Supplementary Figure S7*) was seen and the one-sample t-test for the fits of the *β* for the edges was again significantly different from zero (*p* < 0.0001).

### 3.3 Head position and fingerprinting

The similarity of SNR-group values (derived from head position) across participants for each segment (full-session or split-session) were calculated using Spearman rank correlation (Table 2). Across sessions, correlation values were comparable within-participant and between-participants (0.90 vs 0.89). As a result, identification was low (9%). Within sessions, correlation values within-participant were higher (0.99) compared to between-participants (0.90-0.92) allowing identification rates of 98-100%.

**Table 2:** Head position (SNR-group values): stability across versus within sessions.

	Across-sessions (88 full-sessions)	Within-sessions (176 split-sessions)	
		Preterm (88 splits)	Term (88 splits)
**Within-participant correlation (mean)**	0.90	0.99	0.99
**Between-participants (shared) correlation (mean)**	0.89	0.92	0.90
**Identification Success**	4/44 (9%)	44/44 (100%)	43/44 (98%)

To quantitatively compare the impact of head position (SNR artifact due to head position) with the functional connectivity signal that drives fingerprinting an ordinary least squares multiple linear regression function was used Eq. (2). Both the functional connectivity and SNR-group from the first segment were entered as standardized regressors to predict the functional connectivity in the second segment.

**Fig. 6:**
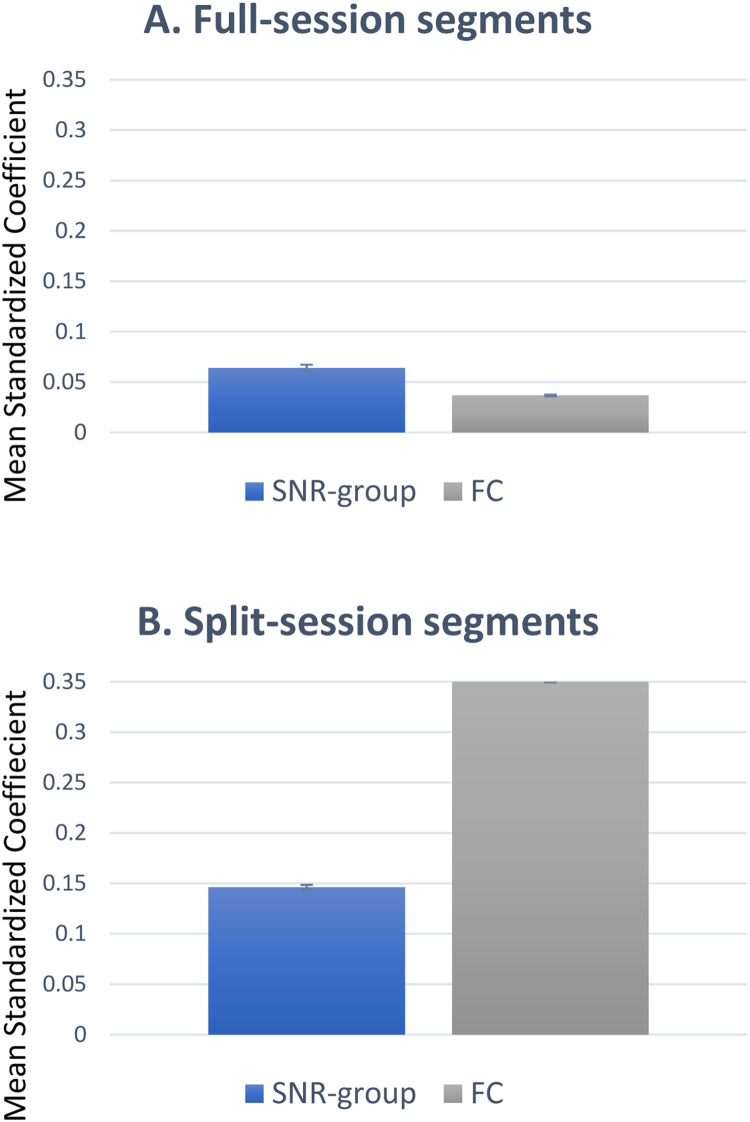
Mean standardized coefficients (95% confidence intervals shown) for: (a) full-session segments (149,381 edges); (b) split-session segments (298,762 edges). Across split-session segments SNR-group accounts for considerable variance.

Fig. 6 shows the mean standardized coefficients (95% confidence intervals marked) for SNR-group and functional connectivity. Across full-session segments (149,381 edges), SNR-group is twice as predictive as functional connectivity. This shows that SNR-group is of critical concern. In split-session segments (298,762 edges) the mean standardized coefficients are higher. Functional connectivity is the strongest predictor (mean 0.35, 95%CI 0.349 – 0.351) but SNR-group still accounts for considerable variance (mean 0.15), at approximately 40% that of functional connectivity.

### 3.4 Factors impacting connectome stability

#### 3.4.1 Individual explanatory variables

Results of the relationship between connectome stability and various individual explanatory variables respectively are shown in Supplementary Sections S3.1 through S3.3 (Figures S 3 to S 5). Head size positively correlated (Pearson r = 0.38, 95%CI 0.08 – 0.62, *p* < 0.016), while inter-session interval (Pearson-r = -0.47 (95% CI -0.67, -0.2), p <0.001) and motion (Pearson r -0.5 (95%CI -0.7, -0.24), *p* = 0.0005) negatively correlated with connectome stability.

#### 3.4.2 Multiple explanatory variables

**Fig. 7:**
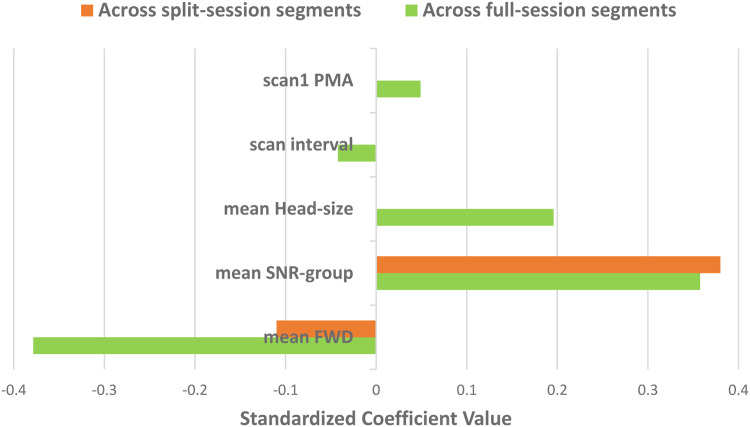
Relative impact of multiple explanatory variables on connectome stability across full-session (5 variables) and split-session (2 variables) segments. [scan1 PMA, preterm session post-menstrual age (weeks); scan interval, interval (weeks) between preterm and term session; mean head-size, mean occipital-frontal circumference (cm) across two full-sessions; mean SNR-group, mean SNR-group (SNR due to head position) across two full/split-session segments; mean FWD, mean framewise displacement (mm) across two full/split-session segments].

Using Eqs. (3) and (4) the relative impact of specific explanatory variables on connectome stability were compared. Fig. 7 shows that, across full-session segments, motion (FWD) is strongly negatively related (β = -0.38, *p* < 0.013) with connectome stability, while SNR-group (SNR based on an individual's head location within the head coil) is positively related (β = 0.36, *p* < 0.05). Across split-session segments (Fig. 7), motion (FWD) was much less related (β = -0.11, *p* < 0.27) to connectome stability compared to head location within the head coil (β = 0.38, *p* < 0.000). In summary, it appears that head position has a considerable relationship to connectome stability, and overall was the strongest factor found, particularly in the across split-session analysis.

## 4 Discussion

The stability of measured connectomes in infants was found to be affected by several factors. The infant's head position relative to the head coil affected the regional SNR, which was empirically found to covary with functional connectivity. Head position alone, in combination with the inhomogeneity of the phased-array head coil, was found to lead to 98-100% success in split-session (within-session) fingerprinting, without any information from an individual's functional connectivity. The significant impact of head position may explain the high fingerprinting identification derived from the within-session method. As a result, the authors of this study propose that within-session fingerprinting must account for this impact, and that care be taken in across-session fingerprinting to control for head position/SNR induced artifacts. Consistent positioning within the head coil across infants is also important.

It was also found that the time interval between sessions negatively correlated with connectome stability, which suggests that the infant connectome may change gradually across time. Furthermore, head motion was found to negatively correlate with connectome stability. Our model of multiple explanatory variables impacting connectome stability showed that head position was the strongest variable found particularly during within-session (across split-session) analysis.

Today fMRI research uses phased array head coils due to their gain of higher SNR in the cortex. However, as the number of head-coil channels increase the inhomogeneity of the distribution of the SNR across the brain also increases, and as a result consistency of head position is imperative for reliable whole-brain functional connectivity analysis and regional functional connectivity estimates. This study has shown how position in native space directly impacts on SNR and that nodes with higher SNR have higher functional connectivity.

Infant head circumference changes rapidly during the first year of life, approximately from 27 cm at 29 weeks gestation (youngest session in this cohort), through 34 cm at term, and 45 cm at 1 year old (50th percentiles) (Department of Health UK and Royal College of Paediatrics and Child Health, 2009). Both a changing head size and head re-positioning (across sessions) therefore impacts on functional connectivity. Across-session identification methods need to be aware of this added variance. Within-session identification methods have less variance due to the constant position of the head and, as a result, identification methods (functional fingerprinting methods) are falsely improved by this confound.

Research groups examining the infant functional connectome continue to use an array of head coils including those with 8-channels (Y. Wang et al., 2021), both 12-channels and 32-channels (Dufford et al., 2021), 32-channels only (Hu et al., 2021) and the dHCP bespoke 32-channels (Ciarrusta et al., 2021; Q. Wang et al., 2021). In the last 10 years or so various groups have looked at designing infant sized head coils. A group in Massachusetts designed custom-sized 32 channel head-coils including for term neonates and 6 month old infants (Keil et al., 2011). These were made to specific set sizes to fit the 95% diameters of these age groups. Similarly another research group designed an 8 channel infant head coil customized to fit the average head of a 6 month old infant (Scheef et al., 2017). Data from the dHCP has been analyzed in this study and recent studies (Ciarrusta et al., 2021; Q. Wang et al., 2021). The dHCP group use a customized infant 32 channel head coil designed using term age infants head sizes (Hughes et al., 2017). To achieve centrality of head position, they use thin inflatable cushion devices to achieve consistent head position within the head coil (Hughes et al., 2017). However, the dHCP infant head coil was not designed to accommodate preterm infant head sizes such as those 44 infants with two sessions (dHCP Data Release 2.0, 2019) whose connectome stability across sessions was analyzed in this study.

Designing head coils that can adapt to the large range of head sizes from preterm through to late infant ages is important for connectome analysis across this time period. In Montreal a research group have tested a pneumatic-based 13 channel infant head-coil that adapts to infant head dimensions (27 weeks PMA to 6 weeks post-term) and which results in a 2.22 fold increase in SNR at the cortex compared to a standard 32 channel adult head-coil (Rios et al., 2018). Most recently a research group in MIT (USA) have designed their own close-fitting 32 channel infant head-coil for ages 1 – 18 months and have tested it both *in vitro* (phantoms) and *in vivo* (infant cohorts) (Ghotra et al., 2021; Kosakowski et al., 2021). Their infant head-coil can be adjusted in the anterior-posterior and lateral dimensions to obtain a snug fit to the infant's head. The tight fit of their infant head coil results in a gain of SNR (2.7 SNR gain in brain regions, compared to adult head coil). These developments in infant head coil design are timely and, for reasons outlined in this paper, this ability to repeatedly approximate the head coil as close as possible to the infant cortex will likely help mitigate much of the variance associated with head position across sessions.

## 5 Conclusion

Research into infant functional connectivity depends heavily on avoiding confounds that are unique or exaggerated in infancy compared to older children/adults. This study has highlighted an important confound namely that infant functional connectome analysis must consider world head position in their design and analysis methods. This study draws attention to the importance of recent developments in head coil design and the emergence of head coils that are adjustable (allowing more closer approximation to the infant cortex). Ideally these head coils should also produce homologous coil coverage.

This study has focused on the effect of head position on functional connectivity measurements in the context of ‘fingerprinting’. However, the head-position induced variation in SNR may have consequences on other measures. For example, it might mask true individual differences in functional connectivity, and add noise or bias to longitudinal measures of functional connectivity during development or at other points of the lifespan. It is hoped that these findings contribute towards both the newly emerging literature on the topic of infant functional connectivity and towards improved methodological practice for longitudinal functional connectivity studies at all age groups.

## Funding sources

This research was supported by an ERC advanced grant 2017 FOUNDCOG 787981.

## CRediT authorship contribution statement

**Graham King:** Conceptualization, Methodology, Formal analysis, Investigation, Visualization, Writing – original draft, Writing – review & editing. **Anna Truzzi:** Formal analysis, Writing – review & editing. **Rhodri Cusack:** Conceptualization, Methodology, Supervision, Funding acquisition, Writing – review & editing.

## Data Availability

Open data from the Developing Human Connectome Project (dHCP) second release was used. Bash scripts and python code are available at GitHub

## Appendix Supplementary materials

Image, application 1
