# Supplementary material for: The confound of head position in within-session connectome fingerprinting in infants
Source: Neuroimage. 2023 Jan;265:119808. doi: 10.1016/j.neuroimage.2022.119808 (PMC9878437; doi:10.1016/j.neuroimage.2022.119808)
Supplement: Supplementary file 1 [file mmc1.docx]

# Proportion of participants contributing towards voxels in the SNR-coil map

We have included a supplementary figure showing the proportion of participants being used in the soft-mean for each voxel in the head coil SNR-coil map (Figure S 1). More centrally all participants contribute towards the SNR-coil, while most peripherally only some of the 416 participants contribute.


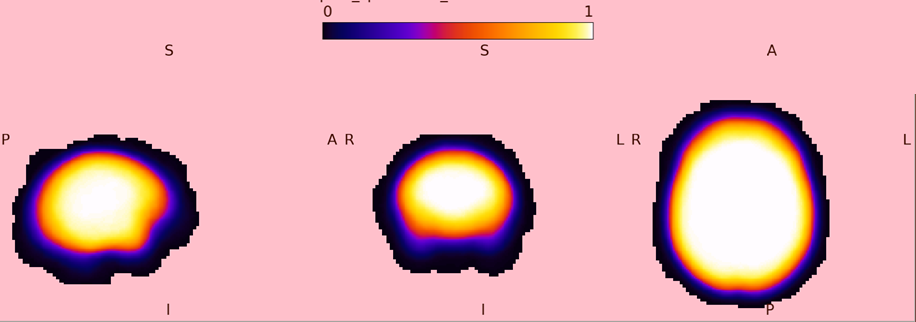


Figure S 1: Shows the proportion (colorbar: 0 – 1) out of total 416 participants used to calculate the soft-mean for each voxel of the SNR-coil map.

# Fingerprinting – a comparison of SNR-group and SNR-individual

In order to compare their ability to mediate fingerprinting we assessed whether SNR-group or SNR-individual in one segment (full-session/split-session) better predicted functional connectivity in another segment. We implemented this using an ordinary least squares multiple linear regression function (<https://www.statsmodels.org/stable/generated/statsmodels.regression.linear_model.OLS.html>).

The standardized regression coefficients this yielded were used to assess predictor strength. The regression model took the form:

Equation S 1

$c_{ij}^{(p,2)}=\beta_{ij}^{SNR}s_{i}^{(p,1)}s_{j}^{(p, 1)}+ \beta_{ij}^{FC}c_{ij}^{(p, 1)} +a$

where

$c_{ij}^{(p,e)}$ is the functional connectivity of the edge between regions $i$ and $j$ for participant $p$ and segment number $e$ (session/split)

$\beta_{ij}^{SNR}$ is the standardized regression coefficient of SNR (SNR-individual/ SNR-group) for the edge

$s{}_{i}^{(p,e)}$ is the SNR value (SNR-individual/ SNR-group) of region $i$ for participant $p$ and segment $e$

$\beta_{ij}^{FC}$ is the standardized regression coefficient of functional connectivity for this edge

$a$ is a constant

SNR (SNR-group/SNR-individual) and functional connectivity were both standardized to obtain z-scores, so that the beta parameters became comparable and indicative of the variance explained. The focus of fingerprinting is on individual differences, and so a separate regression model was conducted for each edge to model the variance across the 44 participants. Regression was repeated for segments comprising two full sessions and for segments comprising two split-sessions. It was repeated in both directions (the dependent variable ($c_{ij}$) being from segment 1 or segment 2). SNR-group and SNR-individual were entered consecutively into Equation S 1.

It can be seen (Figure S 2) that for whole-session segments all coefficients are low with both SNR-group and SNR-individual values comparable to the coefficient for Fc. For split-session segments both SNR-group and SNR-individual coefficients are higher and approximately 40% of the magnitude of the coefficient for Fc. Figure S 2 shows that for both whole-session and split-session segments, SNR-individual had less predictive power for individual differences than SNR-group (SNR-coil map sampled by individual head position). This suggests that coil sensitivity dominates the predictive power of SNR, and that this is best estimated using group-average data.


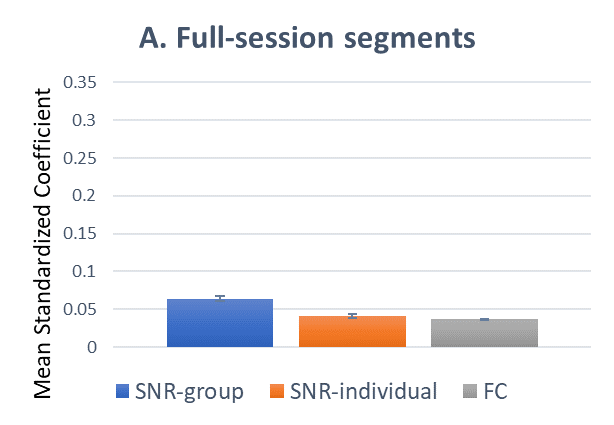

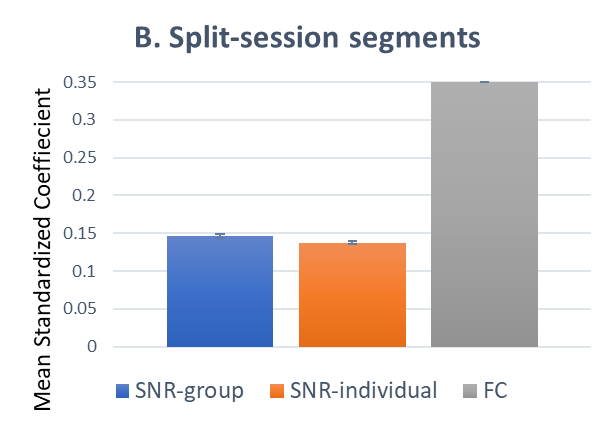


Figure S 2 Mean standardized coefficients (95% confidence intervals) for: a) full-session segments (149,381 edges); b) split-session segments (298,762 edges). SNR-group coefficients are higher than SNR-individual suggesting that coil sensitivity dominates the predictive power of SNR.

# Factors impacting Connectome Stability - Individual explanatory variables

## Connectome Stability and Head Size

Some participants may have a higher overall SNR, because the critical ROIs in the cortex are closer to the coil – for example because their head is larger. An overall higher SNR might be expected to lead a more accurate assessment of the connectome, and greater stability (higher Spearman correlation) across scans. Of the 44 participants with two sessions, 39 infants had head size (occipital frontal circumference, OFC) measured at both sessions. OFC was seen to positively correlate with connectome stability (Pearson r = 0.38, 95%CI 0.08 – 0.62, p<0.016) (Figure S 3).

This correlation was not independent of mean SNR-group across the two sessions (Pearson partial correlation correcting for mean SNR-group was r=0.10, 95%CI -0.23, 0.41, p<0.55). This suggests that a larger head size is associated with increased stability of the connectome and this relationship is likely influenced by a closer approximation of a larger head to the receiver coils (higher SNR).


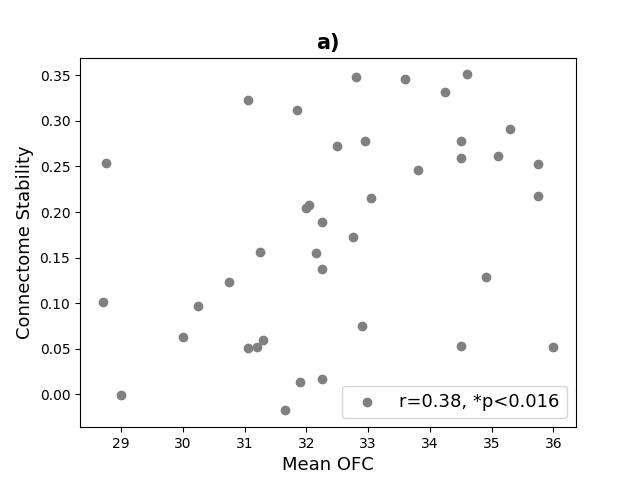


Figure S 3: Functional connectome stability (second order Spearman correlation) versus mean occipital-frontal circumference across two sessions,

## Connectome Stability and Session Interval

The effect of inter-session interval on connectome stability was examined using a scatter plot (Figure S 4, a) with Pearson-r = -0.47 (95% CI -0.67, -0.2), p <0.001. This significant negative correlation indicates that there is also a true component of connectome stability that drifts with development. This negative correlation between inter-session interval and connectome stability was maintained even when correcting for the difference in functional connectivity (difference between preterm and term sessions’ mean connectivity) (Pearson partial correlation r -0.45 (95%CI -0.66, -0.17), p<0.003) indicating that although functional connectivity increases with older post-menstrual age (Figure S 4, b) that the difference in functional connectivity between the preterm and term session is not the explanatory factor for this relationship between inter-session time interval and connectome stability.


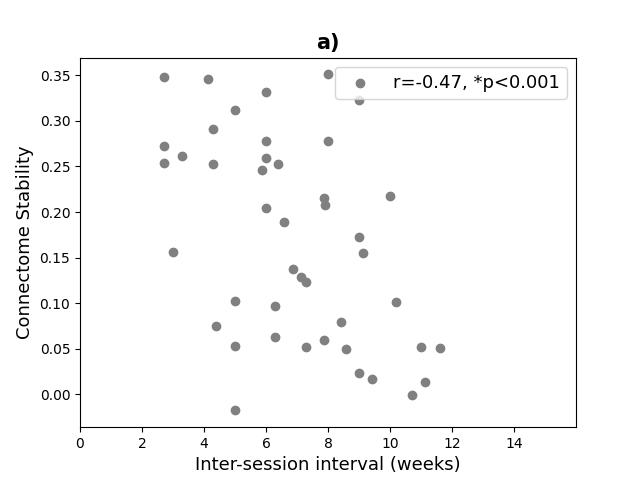

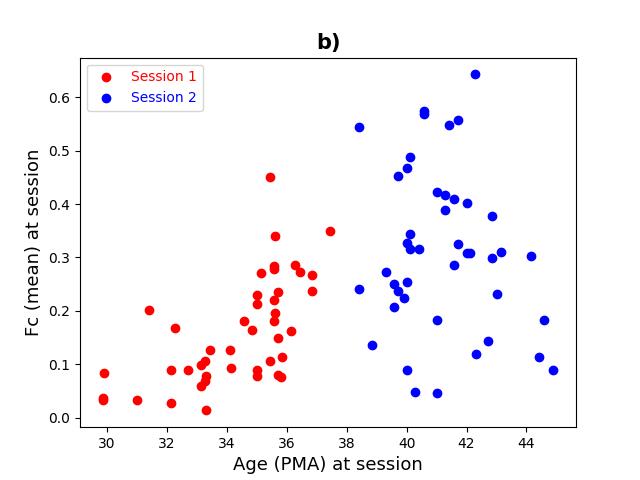


Figure S 4 a) Functional connectome stability (second order Spearman correlation) versus inter-session interval. Preterm connectome stability is negatively correlated with inter-session interval, b) Mean functional connectivity (mean of first order Pearson correlations for 387 ROIs) at session versus post-menstrual age (PMA) at session

## Connectome Stability and Motion

The dHCP cohort was scanned without sedation and so has large motion artifact (Fitzgibbon et al., 2020). As a result, the dHCP pre-processing pipeline does not perform motion censoring in order to minimize the potential loss of participants or timepoints. Instead, the dHCP use a ‘less aggressive’ ICA-based denoising procedure and authors conclude that this ICA-based denoising procedure is qualitatively similar to using spike regression in periods of time when motion is worst (Fitzgibbon et al., 2020).

Connectome stability (across sessions) in this preterm population was negatively correlated with mean framewise displacement (Pearson r -0.5 (95%CI -0.7, -0.24), p = 0.0005) (Figure S 5) This negative correlation was independent of mean SNR-group values (mean across two sessions) (Pearson partial correlation: r -0.4 (95%CI -0.62, -0.11) p<0.008) indicating that head motion has a negative effect on connectome stability that is independent of SNR-group (SNR due to head position in the head coil space).


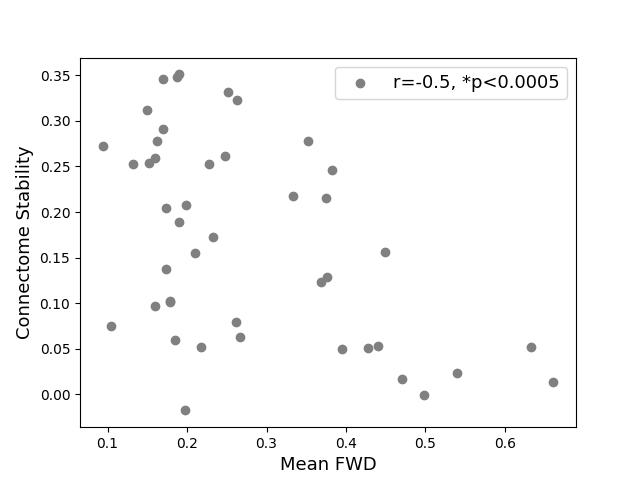


Figure S 5 Connectome stability versus mean framewise displacement (mean of the FWD across two sessions). Preterm connectome stability is negatively correlated with head motion.

# Relationship between head position (SNR-group values) and functional connectivity


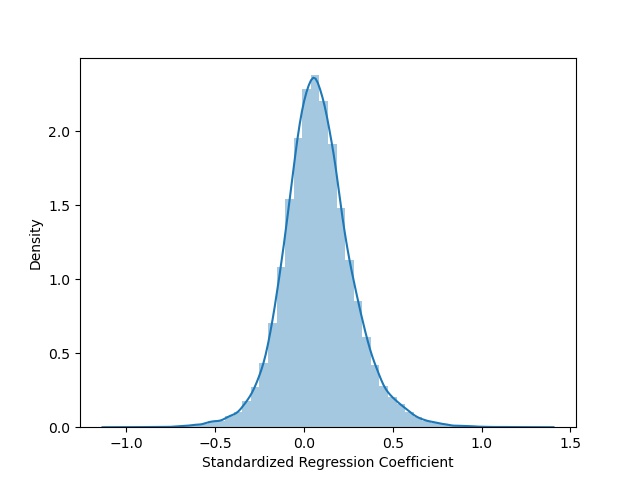


Figure S 6: Histogram of standardized coefficient values (slopes) of 74,691 edges (linear regression across 416 participants with one session) for the relationship between functional connectivity and SNR due to head position (SNR-group). Mean coefficient = 0.08


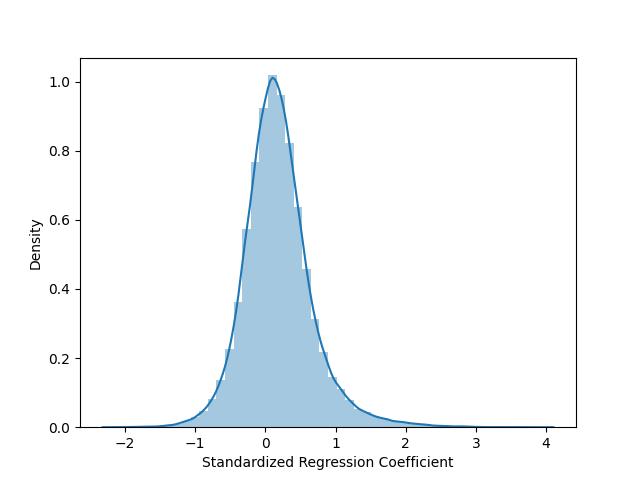


Figure S 7: Histogram of standardized coefficient values (slopes) of 74,691 edges (linear regression across independent group of 44 participants with both a preterm and term session) for the relationship between functional connectivity and SNR due to head position (SNR-group). Mean coefficient = 0.18
